# Long-term efficacy of subtotal splenectomy due to portal hypertension in cirrhotic patients

**DOI:** 10.1186/s12893-015-0077-2

**Published:** 2015-07-24

**Authors:** Haibo Chu, Wei Han, Lei Wang, Yongbo Xu, Fengguo Jian, Weihua Zhang, Tao Wang, Jianhua Zhao

**Affiliations:** Center of General Surgery, The 89th Hospital of People’s Liberation Army, West Beigong Road 256, Weifang, 261021 China; Department of Postgraduate, Weifang Medical University, Weifang, 261042 China; Department of General Surgery, Changyi People’s Hospital, Changyi, 261300 China; Department of General Surgery, Weifang Traditional Chinese Medicine Hospital, Weifang, 261041 China; Department of Pathology, The 89th Hospital of People’s Liberation Army, Weifang, 261021 China

**Keywords:** Portal hypertension, Splenomegaly, Subtotal splenectomy, Shunt

## Abstract

**Background:**

Portal hypertension (PHT) requires invasive measures to prevent rupture and bleeding of esophagogastric varices; however, the long-term results of subtotal splenectomy plus fixation of the retrosternal omentum majus (SSFROM) have not been reported. Specifically, the advantages and disadvantages of surgery that preserves the spleen and the long-term hematologic effects have not been described.

**Study design:**

Our studies relating to SSFROM commenced in February 1999. As of April 2014 we have performed 256 subtotal splenectomies The records of 65 patients with PHT who underwent SSFROM were reviewed retrospectively.

**Results:**

Four patients died within 4 years of surgery, with a 4-year survival rate of 94 %; the 11-year survival rate was 60 %. Eleven patients (17 %) had re-bleeding from esophagogastric varices. The white blood cell and platelet counts were higher 6 and 11 years post-operatively compared with pre-operative values (*P* < 0.01). Portal venous diameter, portal venous flow volume, splenic artery flow volume, as well as splenic length, thickness, and average cross-sectional areas were shown to be significantly constricted or decreased (*P* < 0.01). The proportion of serum CD3+ T cells, CD4+ T cells, and CD8+ T cells was increased (*P* < 0.01), while the serum levels of macrophage colony-stimulating factor and granulocyte-macrophage colony-stimulating factor were significantly decreased (*P* < 0.01). There was no significant change in the serum levels of IgA, IgM, IgG, and Tuftsin (*P* > 0.05). DSA demonstrated that 15 cases formed collateral circulations between the portal vein and superior vena cava.

**Conclusion:**

SSFROM provide long-term hemostasis for esophagogastric variceal bleeding in PHT and corrected hypersplenism. SSFROM is an effective treatment for patients with PHT in whom long-term survival is expected.

## Background

The incidence of portal hypertension (PHT) with post-hepatitis cirrhosis is higher in China than elsewhere worldwide. PHT with concurrent esophagogastric variceal bleeding is one of the common causes of early death [1]. Vein ligation under endoscopy and injection sclerotherapy are the preferred hemostasis methods, and bleeding control rates are 89.7 and 87.5 %, respectively [2, 3]. A switch to decompressive shunt procedures is mandatory if endoscopic therapy fails to control recurrent variceal hemorrhage [4]. We rely on bionics to devise a new technique to treat PTH with subtotal splenectomy plus fixation of the retrosternal omentum majus (SSFROM) [5]; however, this technique is controversial. In the present study, the re-bleeding rate, survival rate, hemodynamics, hematologic parameters, splenic function, immunologic parameters, and related indices were analyzed retrospectively to evaluate the long-term efficacy of SSFROM in patients with PHT.

## Methods

Ethical approval of the study protocol (No. 1685) was obtained from the Human Research Ethics Committee of the 89^th^ Hospital of PLA (Weifang, China). All individuals provided written informed consent before enrollment in the study.

### Study population

Our studies relating to SSFROM commenced in February 1999. As of April 2014 we have performed 256 subtotal splenectomies (preserving the lower pole, normal spleen size of splenic tissue, splenic omentum, and splenocolic vessels for the purpose of blood supply). Sixty-five patients (34 males and 31 females; mean age, 34 years; age range, 25–43 years) with splenomegaly due to PHT were randomly selected; 13 patients underwent emergency surgery and 52 patients underwent elective surgery. Liver biopsies were performed on all patients to confirm cirrhosis. Twenty-three patients had bleeding episodes prior to surgery and underwent endoscopic injection sclerotherapy pre-operatively. Six patients underwent six sessions of endoscopic injection sclerotherapy. Patients were confirmed to have cirrhosis after hepatitis-B infection, HBV-DNA was negative, and were class A or B according to the Child–Pugh classification. Cirrhosis was accompanied by hypersplenism with moderate or severe varicose veins of the lower esophagus, plus a history of digestive tract hemorrhage. All patients were followed up continuously.

### Operative method

**Fig. 1:**
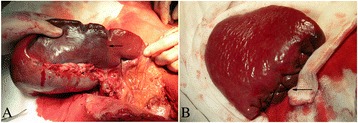
**a** The lienorenal and splenophrenic ligaments were ligated. then, the gastrosplenic ligament and splenic artery stem were ligated. The ischemic separatrix occurred obviously in spleen (arrow). **b** The residual splenic section was sutured using a horizontal mattress cross-suture method (arrow)

A left upper abdominal “L” incision was made in the abdomen to explore the liver and spleen. First, the lienorenal and splenophrenic ligaments were ligated. Then, the spleen was moved through the incision. Second, the gastrosplenic ligament and splenic artery stem were ligated. The left gastroepiploic and splenocolic vessels were preserved (Fig. 1a). Third, along the ischemic separatrix the subtotal spleen was removed; the spleen bed was sutured by serous membranization. Residual spleen, 11 × 7 × 4 cm in size, was preserved. The residual splenic section was sutured using a horizontal mattress cross-suture method (Fig. 1b). Fourth, the splenic capsule was cauterized with an electrotome to create a rough surface. Pericardial devascularization was performed. Fifth, the omentum majus (10 × 5 cm and 10 × 10 cm) was excised and the retrosternal space was separated. A part of the omentum majus was tamponaded into the retrosternal space and fixed in place. The residual spleen and the other omentum majus were fixed into the retroperitoneum. Drainage catheters were placed in the splenic recess, and the abdominal cavity was closed.

### Evaluation

The cumulative survival and non-bleeding rates were determined by the Kaplan–Meier method. The portal venous diameter, portal venous flow volume, and splenic artery flow volume were detected by colour Doppler sonography. The peripheral white blood cell and platelet counts were measured using a hematology analyzer. An abdominal CT scan measured the splenic size (splenic length, splenic thickness, and splenic square area). Splenic scintigraphy with ^99m^Tc sulfur colloid evaluated the phagocytic function of the residual spleen by ECT. The serum CD3+ T cells, CD4+ T cells, and CD8+ T cells were enumerated by flow cytometry. Serum macrophage colony-stimulating factor (M-CSF) and granulocyte-macrophage colony-stimulating factor (GM-CSF) were measured by ELISA. Serum Tuftsin was measured with a radioimmunoanalyzer. Serum IgA, IgM, and IgG were measured with a fully automatic biochemical immune analyzer. Using digital subtraction angiography (DSA) through an indwelling catheter in vessels of the omentum majus post-operatively, the collateral vascular shunt between the portal vein and superior vena cava was observed.

### Statistical analyses

Data analyses were performed using SPSS (version 17.0; SPSS, Inc., Chicago, IL, USA). All values are the mean ± standard deviation. Groups were compared using a t-test. A *P* < 0.05 was considered significant.

## Results

### Survival rate

**Fig. 2:**
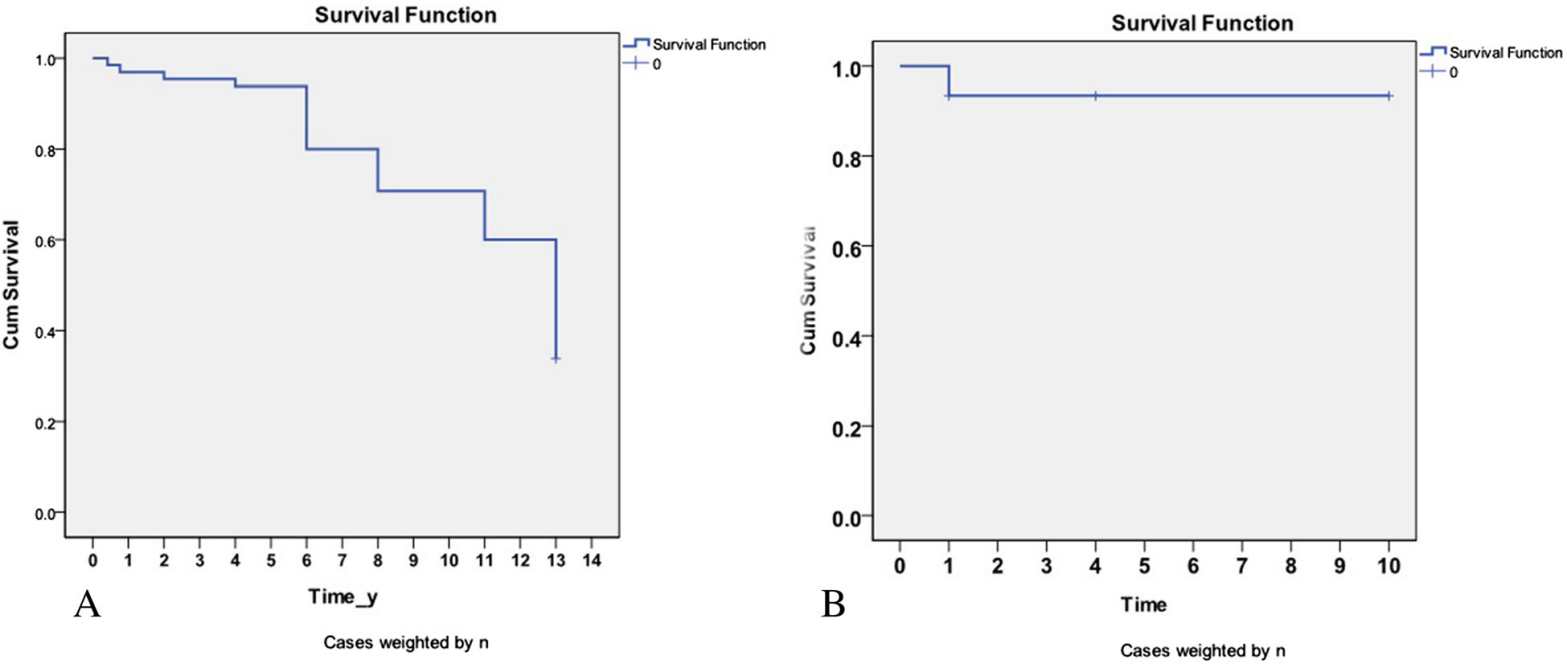
**a** Cumulative survival rates after subtotal splenectomy plus fixation of the retrosternal omentum majus (SSFROM) for portal hypertension (PHT). The 4- and 11-year survival rates were 94 and 60 %. **b** Cumulative nonbleeding rates after SSFROM for PHT. Postoperative recurrent bleeding ccurred in eleven patients (17 %). The 4- and 10-year non-bleeding rates were both 83 %

The 4- and 11-year survival rates were 94 (61/65) and 60 % (39/65), respectively. Four patients died. Two patients survived for 152 and 278 days. The cause of death was complete occlusion of the portal vein as a result of portal thrombosis. Two patients survived for 2 and 4 years. The cause of death was hepatic carcinoma complicating liver failure (Fig. 2a).

### Recurrent bleeding rate

**Fig. 3:**
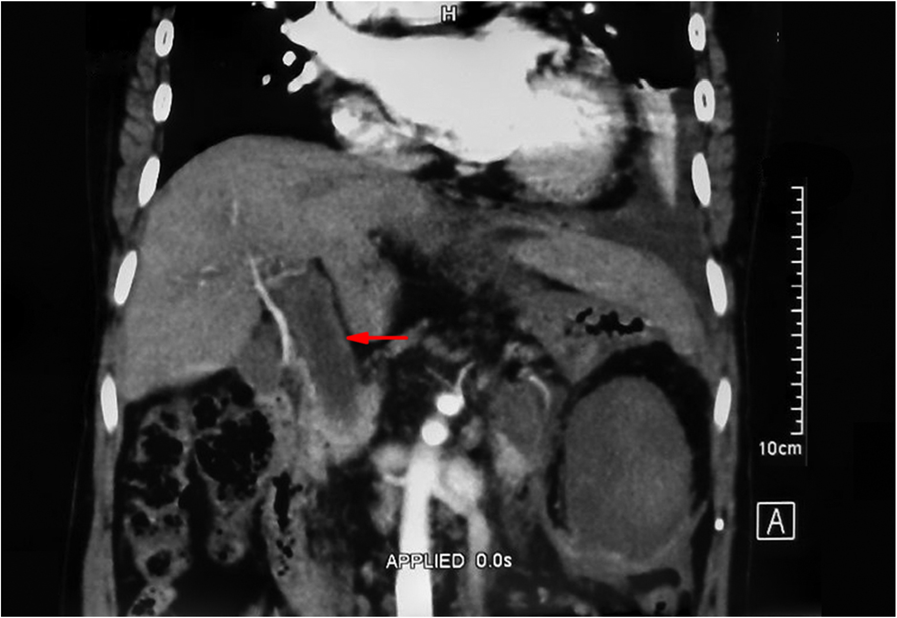
CT scan showed that patients was complicated with portal thrombosis at 4.5 months post-operatively

Post-operative recurrent bleeding occurred in 11 patients (17 %), due to esophageal varices in eight patients and from a gastric varix in three patients. All of the patients were cured with conservative therapy. Of the 11 patients who bled post-operatively, seven had a history of pre-operative bleeding and four were complicated with portal thrombosis post-operatively. The 4- and 10-year non-bleeding rates were both 83 % (54/65; Fig. 2b). No patients have died from esophagogastric variceal bleeding to data. Post-operative endoscopy of the stomach showed exacerbation of esophagogastric varices in 11 patients and prophylactic endoscopic injection sclerotherapy was performed, after which neither re-bleeding nor re-exacerbation occurred. Portal thrombosis occurred in six patients (9 %), from pre-operative partial portal thrombosis and post-operative exacerbation to complete occlusion of the portal vein in two patients (Fig. 3).

### Change in peripheral blood cells

**Fig. 4:**
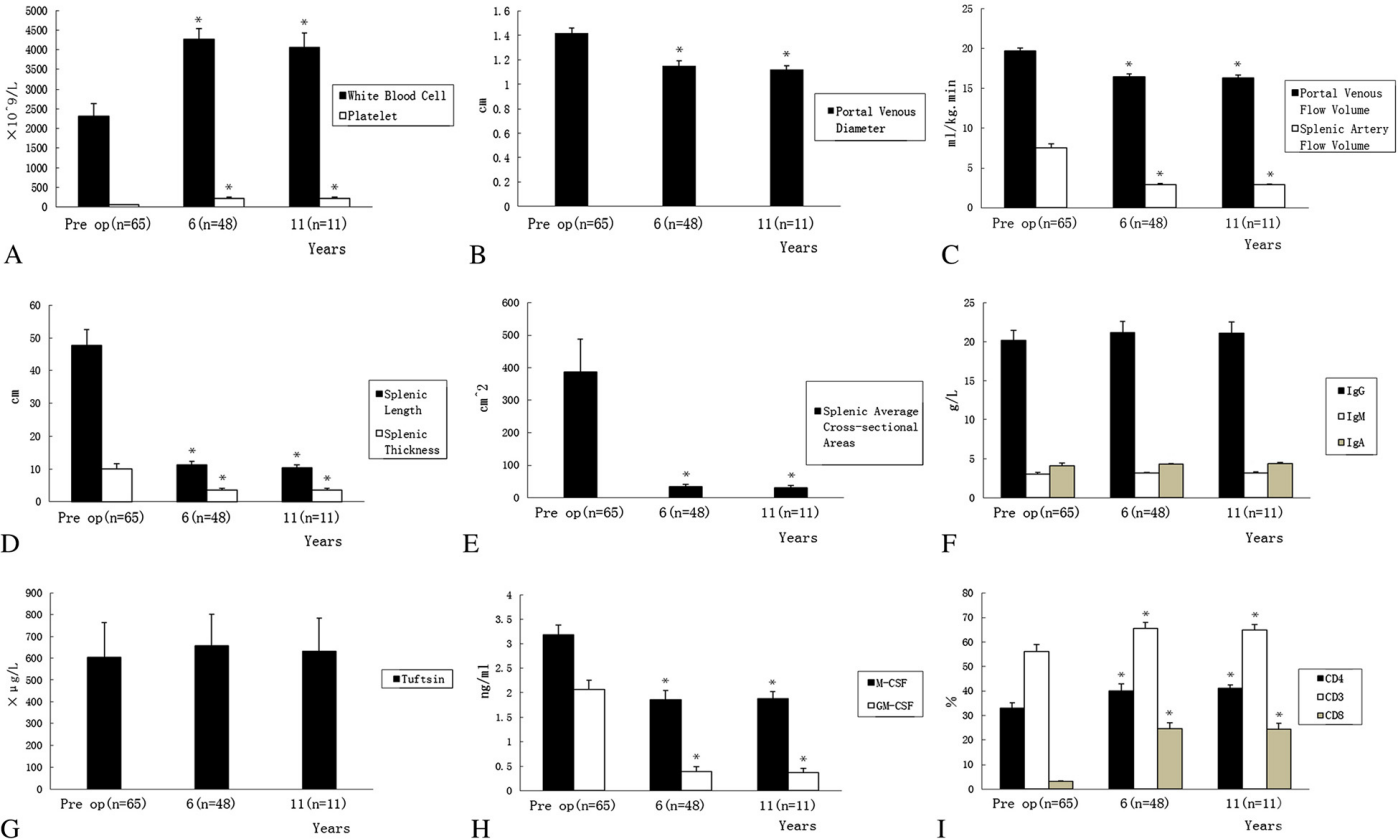
a Changes in the white blood cell (WBC) count and platelet count at 6- and 11- years, after SSFROM for PHT (*P < 0.01). **b** Change in portal venous diameter at 6- and 11- years, after SSFROM for PHT (*P < 0.01). **c** Changes in portal venous flow volume, and splenic artery flow volume at 6- and 11- years, after SSFROM for PHT (*P < 0.01). **d** Changes in splenic length and splenic thickness at 6- and 11- years, after SSFROM for PHT (*P < 0.01). **e** Change in splenic square area. at 6- and 11- years, after SSFROM for PHT (*P < 0.01). **f** Changes in levels of serum IgA, IgM and IgG at 6 and 11 years, after SSFROM for PHT (*P > 0.05). **g** Change in levels of serum Tuftsin at 6 and 11 years, after SSFROM for PHT (*P > 0.05). **h** Changes in levels of serum macrophage colony-stimulating factor (M-CSF) and granulocyte-macrophage colony-stimulating factor and (GM-CSF) at 6 and 11 years, after SSFROM for PHT (*P < 0.01). **i** Changes in proportion of serum CD3+ T cells, CD4+ T cells, and CD8+ T cells at 6 and 11 years, after SSFROM for PHT (*P < 0.01)

The white blood cell and platelet counts were associated with a significant rise 6 years post-operatively in 48 patients and 11 years in 16 patients post-operatively (*P* < 0.01; Fig. 4a).

### Change in hemodynamics of the splenic artery and portal vein

There were significant differences between the pre- and post-operative portal venous diameter, portal venous flow volume, and splenic artery flow volume (*P* < 0.01; Fig. 4b, c).

### Change in splenic volume

There were significant differences between the pre- and post-operative splenic length, splenic thickness, and splenic square area (*P* < 0.01; Fig. 4d, e).

### Change in immunologic and related indices

There were no significant differences between the IgA, IgM, IgG, and Tuftsin levels pre- and post-operatively (P > 0.05; Fig. 4f, g). The serum levels of M-CSF and GM-CSF were decreased significantly, and there was a significant difference between the pre- and post-operative values (*P* < 0.01; Fig. 4 h). The proportion of CD3+ T cells, CD4+ T cells, and CD8+ T cells was increased post-operatively compared with pre-operative values (*P* < 0.01; Fig. 4i).

### Change in splenic function

**Fig. 5:**
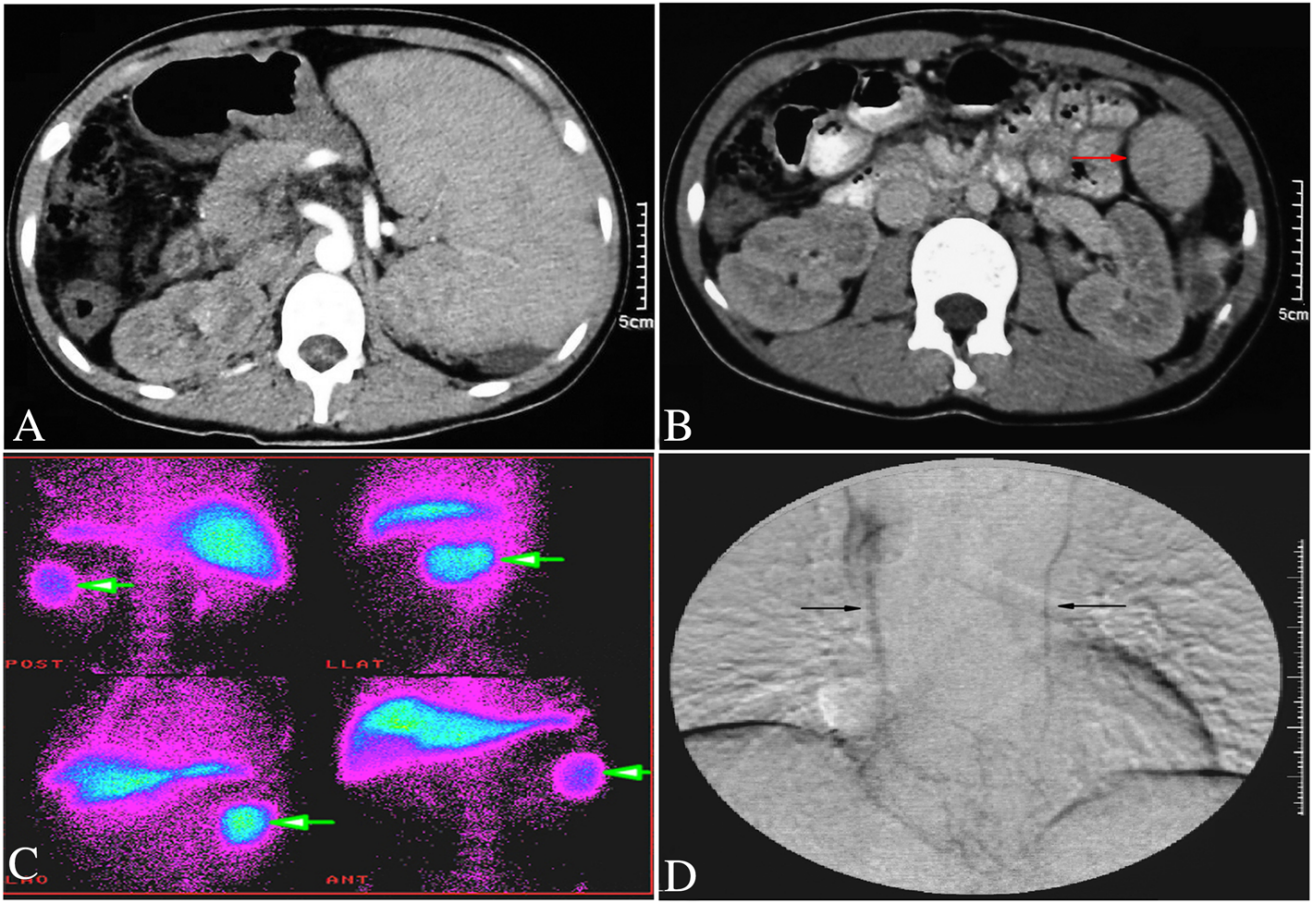
**a** CT scan revealed splenomegaly pre-operatively. **b** CT scan revealed that the blood supply of the residual spleen was normal (arrow) at 1-year, after SSFROM for PHT. **c** Isotope scanning with 99mTc showed that the residual splenic phagocytic function had recovered (arrow) at 4-years, after SSFROM for PHT. **d** DSA showed an established collateral circulation between the portal vein and superior vena cava (arrow) at 1 month, after SSFROM for PHT

A CT scan revealed that the blood supply of the residual spleen was normal (Fig. 5a, b). Isotope scanning with ^99m^Tc showed that the residual splenic phagocytic function had recovered (Fig. 5c).

### Portal-azygous collateral circulation

Fifteen patients had indwelling catheters placed in the omental vein 1 month post-operatively. DSA showed that omental venous blood flowed into the superior vena cava. These results confirmed an established collateral circulation between the portal vein and superior vena cava (Fig. 5d).

## Discussion

PHT is a clinical syndrome caused by the continual rise in portal vein pressure, and is closely associated with the incidence and mortality of chronic liver disease. The severity of PHT is indicated by a hepatic portal venous pressure gradient (difference between hepatic sinal portal wedge pressure and free hepatic venous pressure) [6]. Under high pressure, reactive changes are observed in the intra- and extra-hepatic vasculature, such as shear stress, vascular proliferation, and neovascularization. Collateral circulation plays an important role in buffering portal pressure [7]. Portal-systemic collateral vascular resistance and vasoconstrictor responsiveness are crucial in PHT and control of variceal bleeding [8]. PHT induces the formation of portal systemic collaterals. Revealing the developmental change in portal-systemic collaterals is important for future therapy [9]. The incidence of gastric varices is 25 % and the rate of gastric variceal hemorrhage is less than esophageal variceal hemorrhage [10]. As PHT develops, the formation of collateral vessels and arterial vasodilation progresses, which results in increased blood flow to the portal circulation [11]. The special anatomic structure of the esophageal venous plexus results in easy, and even fatal, variceal bleeding. According to statistics, esophageal varices are detected in approximately 50 % of cirrhosis patients, and 5–15 % of cirrhotic patients have newly formed varices or worsening of varices each year [12]. Thus, an understanding of the pathogenesis of the collateral circulation and compensatory mechanism in patients with PHT is crucial.

The spleen is the only solid organ of the portal venous system. In patients with PHT, portal venous blood returns flow and the splenic functions as an auxiliary capacitance system which compensates for the increase in portal pressure that may cause splenomegaly [13]. The splenic angiogenesis can regulate the portal systemic collateral circulation and increase splenic blood flow, which is a pathophysiologic hallmark of the spleen [14, 15]. Traditionally, splenomegaly has been thought to be due to passive splenic congestion. Recent research suggests that factors of splenomegaly in portal hypertension include not only spleen congestion, as traditionally thought, but also enlargement and hyperactivation of the splenic lymphoid tissue, as well as increased angiogenesis and fibrogenesis. Splenomegaly will not occur if the spontaneous portal systemic shunt exists. Therefore, portal systemic collaterals and splenomegaly are the compensation of PHT. Of portal hypertensive patients with splenomegaly and hypersplenism, 64 % have a simultaneous decrease in various blood cells, 36 % have a decrease in single type blood cells, and 52 % of patients have bone marrow hyperplasia. Therefore, a decrease in peripheral blood cells and bone marrow hyperplasia are crucial indices of hypersplenism [16, 17]. In splenomegaly, the medial ramus of perisplenic ligaments (lienorenal, splenophrenic, and splenocolic ligaments) have abundant vessels, and extensive collateral circulation is established between the retroperitoneal space and pericardium, thus the spleen can be called a “shunt bridge”. The major innovation of the Warren operation utilizes the spleen as a shunt bridge, which shunts blood flow to the stomach and spleen to decrease pressure selectively [18].

In general, vein ligation under endoscopy and injection sclerotherapy are the preferred hematostatic method for esophagogastric variceal bleeding, which have the advantages of minimal trauma and rapid control of hemorrhage compared with surgery; however, for some patients, the long-term recurrent bleeding rate is higher than surgery [19, 20]. For surgeons, the portosystemic shunt should be performed after a transjugular intrahepatic portosystemic shunt and before a liver transplantation [21]. In a study by *Orloff et al*., [22] a portal-systemic shunt was used to treat 200 patients with portal vein thrombosis (extrahepatic PHT). Actuarial 5-, 10-, and 15-year post-operative survival rates were 99 %, 97 %, and 95 %, respectively. No patient developed portal-systemic encephalopathy. *Gajin et al*., [23] compared different surgeries in PHT patients and found that in the group of patients treated by distal splenorenal shunt with partial spleen resection, only one patient had splenomegaly post-operatively (5 %), while in the group of patients treated by a distal splenorenal shunt only, there were 13 patients with splenomegaly (68 %). There was a significant statistical difference in platelet count and splenic volume, which revealed that distal splenorenal shunt (Warren) plus partial spleen resection was superior to the original Warren. *Sretenovic et al*., [24] performed distal splenorenal shunts plus partial resection of the spleen in 16 children with extrahepatic PHT. The post-operative 2-year recurrent bleeding rate was 5 % and the incidence of thrombosis was 7 %. This surgical technique was applicable to patients with extrahepatic PHT. Because of favorable liver function and without cirrhosis, the post-operative long-term survival rate was higher and the recurrent bleeding rate was lower. However, for patients with post-hepatitis cirrhosis, liver cancer and liver failure may occur due to poor liver function and the long-term survival rate decreased. However, for cirrhosis after hepatitis B infection, because of unfavorable liver function, there was an increased probability for hepatomas and liver failure, and the post-operative long-term survival rate decreased. Therefore, the survival and recurrent bleeding rates depended on liver function.

SSFROM include the following. 1. Subtotal splenectomy: The short gastric vessels and splenic artery stem were ligated, preserving the lower splenic pole, and the residual spleen was supplied by the left splenic omentum and splenocolic vessels. The residual spleen and partial omentum were fixed into the retroperitoneum. On the one hand, the residual spleen can eliminate the high-pressure state, and prevent recurrence of splenomegaly and hypersplenism. On the other hand, as a bridge of the portal-systemic shunt, the residual spleen can shunt the high-pressure venous flow into the systemic circulation. 2. Pericardial devascularization: Hemostasis of vascular devascularization, especially the high esophageal branch, which was crucial before the portal-systemic shunt was established. 3. Fixation of the retrosternal omentum majus: Collateral circulation was established between the omentum majus and azygos vein; this decreases the bleeding risk of a natural portal-azygous collateral pathway. Shunt flow volume can follow up the portal pressure gradient and thoracic coeliac pressure difference in self-regulation, without concern about stenosis of the anastomotic stoma and embolization occurring. Post-operative DSA showed that the portal-azygous collateral circulation was established. Color Doppler ultrasound suggested that portal blood flow volume and portal venous diameter have a significant constriction or decrease compared with pre-operatively. The recurrent bleeding rate was 17 % without hepatic encephalopathy. It was revealed that this surgical technique has the long-term effect of shunting, decreased pressure, and controlled bleeding. The long-term follow-up of patients after subtotal splenectomy showed that residual splenic blood flow decreased; no residual splenic volume enlargement occurred. Radioisotope scanning showed that residual splenic phagocytosis was normal. Residual splenic immune indices were the same as pre-operatively. Post-operative peripheral white blood cell and platelet counts were associated with a significant pre-operative rise. Not only was the residual splenic blood flow reduced, but hypersplenism was corrected and residual splenic phagocytosis and immune function still existed. The level of serum T lymphocyte subpopulation was higher and the level of serum M-CSF and GM-CSF was lower post-operatively compared with pre-operatively. The results suggested that residual splenic tissue of relieving high pressure can enhance the immune reaction and weaken the inflammatory reaction. Our previous study showed that there was no significant difference in the content of collagen, elasticity, and reticular fiber between the residual spleen and splenomegaly. Electron microscopy showed that the ultrastructure of lymphocytes was improved. Immunohistochemical staining showed that counts per unit area of T and B lymphocytes and macrophages increased markedly post-operatively compared with pre-operatively [25, 26]. Thus, it can be seen that the anatomic and physiologic foundation of subtotal splenectomy provides valuable clinical information for splenic preservation. Our follow-up results (recurrent bleeding rate, and hemodynamic, hematologic, and immunologic indices) were nearly in agreement with that reported by *Hase et al* [27]. There is a difference in survival rate, which may be caused by different liver function. Hemorrhoidal bleeding was not observed in our study. Our data showed that the major reasons for massive post-operative bleeding were as follows: 1. In the short-term, collateral circulation was not established between the portal and azygos veins, and the portal venous pressure of the patients after disconnection showed a transient increase. and 2. After disconnection, blood flow to the lower esophagus and gastric wall had a high dynamic state, which induced a submucosal arteriovenous shunt, mucosal ischemia, hypofunction of the barrier, and formed the pathogenic basis of mucosal lesions. Our data also showed that the incidence of post-operative portal thrombosis was 9 %, which was lower than other reports [28–32]. The analyses were related to protection of the partial spleen and administration of a corresponding anticoagulant measure(low-molecular weight heparin calcium [5000 U subcutaneously] starting on the 2nd day after surgery (daily for 2 weeks). Then, warfarin or rivaroxaban was taken orally once a day for 3 months). In the current study, the patients with post-operative portal thromboses were treated with anticoagulation and antiagglutinating as well as thrombolysis, and was absorbed completely or the improvement of absorption within 2-4 weeks. In our opinion, compared with the Warren operation, SSFROM is a simple operation, is easy to popularize, and can obtain the same clinical effect; therefore it is worth popularizing. However, it must be emphasized that the patients in this study were select patients, class A or B according to the Child–Pugh classification, and controlled content of the virus can improve the long-term survival rate.

## Conclusion

SSFROM provides long-term hemostasis for esophagogastric variceal bleeding in PHT and corrects hypersplenism. SSFROM is an effective treatment for patients with PHT in whom long-term survival is expected.

## Abbreviations

PHT: Portal hypertension
SSFROM: Subtotal splenectomy plus fixation of the retrosternal omentum majus
M-CSF: Macrophage colony-stimulating factor
GM-CSF: Granulocyte-macrophage colony-stimulating factor
DSA: Digital subtraction angiography
